# Differential anti-tumour effects of MTH1 inhibitors in patient-derived 3D colorectal cancer cultures

**DOI:** 10.1038/s41598-018-37316-w

**Published:** 2019-01-28

**Authors:** Lizet M. van der Waals, Jamila Laoukili, Jennifer M. J. Jongen, Danielle A. Raats, Inne H. M. Borel Rinkes, Onno Kranenburg

**Affiliations:** Laboratory Translational Oncology, UMC Utrecht Cancer Center, Utrecht, The Netherlands

## Abstract

Reactive oxygen species (ROS) function as second messengers in signal transduction, but high ROS levels can also cause cell death. MTH1 dephosphorylates oxidized nucleotides, thereby preventing their incorporation into DNA and protecting tumour cells from oxidative DNA damage. Inhibitors of MTH1 (TH588 and *(S)*-crizotinib) were shown to reduce cancer cell viability. However, the MTH1-dependency of the anti-cancer effects of these drugs has recently been questioned. Here, we have assessed anti-tumour effects of TH588 and *(S)*-crizotinib in patient-derived 3D colorectal cancer cultures. Hypoxia and reoxygenation – conditions that increase intracellular ROS levels – increased sensitivity to *(S)*-crizotinib, but not to TH588. *(S)*-crizotinib reduced tyrosine phosphorylation of c-MET and ErbB3 whereas TH588 induced a mitotic cell cycle arrest, which was not affected by adding ROS-modulating compounds. Furthermore, we show that both compounds induced DNA damage that could not be prevented by adding the ROS inhibitor N-acetyl-L-cysteine. Moreover, adding ROS-modulating compounds did not alter the reduction in viability in response to TH588 and (*S*)-crizotinib. We conclude that TH588 and (*S*)-crizotinib have very clear and distinct anti-tumour effects in 3D colorectal cancer cultures, but that these effects most likely occur through distinct and ROS-independent mechanisms.

## Introduction

In general, cancer cells produce relatively high levels of reactive oxygen species (ROS), and this is a phenomenon that could be exploited therapeutically^1–3^. We recently showed that colorectal cancer (CRC) liver metastases are characterized by higher levels of oxidative stress compared to corresponding primary tumours^4^. Furthermore, ROS neutralizing capacity is essential for the establishment of distant metastases^5–7^. Metastasis formation and tumour recurrence after therapy are often associated with cycles of regional hypoxia and reoxygenation, which causes the production of high levels of ROS, and may provide a tumour vulnerability. Therapeutics that interfere with redox signalling and further enhance oxidative damage under hypoxic conditions and during reoxygenation, may thus be effective in preventing hypoxia-associated tumour growth, metastasis formation and recurrence.

TH588^8^ and *(S)*-crizotinib^9^ are inhibitors of the mutT homologue 1 (MTH1) phosphatase. MTH1 dephosphorylates oxidized nucleosides, thereby preventing their incorporation into DNA^10,11^ (Reviewed by^12^). MTH1 inhibitors may exploit high ROS levels in cancer cells as a generic phenomenon that is associated with tumorigenesis, to selectively target cancer. Interestingly, hypoxia enhanced the mRNA and protein expression of MTH1 in two CRC cell lines^13^. Indeed, the functional requirement for MTH1 is likely to be highest under conditions of chronically-elevated ROS levels, resulting in high levels of MTH1 substrates^14^. However, whether MTH1 is indeed a valid target for anti-cancer therapy has recently been questioned^15–19^. Moreover, the MTH1-dependency of the anti-tumour effects of TH588 and *(S)*-crizotinib has also been challenged^15–19^.

TH588 may (partly) exert its function via disruption of microtubule networks and inducing a mitotic cell cycle arrest^16^. *(S)*-crizotinib is the optical isomer of a clinically approved anticancer agent, called xalkori or *(R)*-crizotinib, a drug known to inhibit c-MET activity (also known as the hepatocyte growth factor receptor). *(S)*-crizotinib also reduces phosphorylation of c-MET, although with reduced efficiency compared to *(R)*-Crizotinib^20^. The MTH1 inhibitory potency of *(S)*-crizotinib is much higher than that of *(R)*-crizotinib^9,21^. Surprisingly, proteomics analysis of drug responses showed that *(S)*-crizotinib did not cluster with five other MTH1 inhibitors^16^.

Thus, while the interest in TH588 and (*S*)-crizotinib as anti-cancer therapeutics is increasing, much is still unclear about their optimal application and their mechanism of action. This prompted us to assess the anti-tumour effects of TH588 and *(S)*-crizotinib head-to-head in patient-derived 3D CRC cultures. We compared normoxia and hypoxia/reoxygenation, conditions that increase ROS levels^3^, impose an aggressive cancer phenotype^22,23^ and promote drug resistance (as reviewed by^24^). To assess ROS-dependency of the observed effects, TH588 and (*S*)-crizotinib treatment were combined with ROS-modulating compounds. It is of major importance to establish whether both compounds are dependent on ROS to exert their function. If these compounds exploit increased ROS signalling in tumour cells they might have limited side effects, which could increase their clinical potential.

We found that *(S)*-crizotinib blocked phosphorylation of c-MET and Erb-B2 receptor tyrosine kinase 3 (ErbB3), whereas TH588 induced a mitotic cell cycle arrest, similar to nocodazole. Hypoxia and reoxygenation increased sensitivity to *(S)*-crizotinib, but not to TH588. However, combined treatment with ROS-modulating agents did not influence the anti-tumour effects of both compounds. Our findings thus show that both compounds have clear anti-cancer effects in 3D CRC models, but also that these effects are distinct and, most likely, independent of ROS.

## Results

### TH588 and (*S*)-crizotinib reduce cell viability of patient-derived 3D CRC cultures

**Figure 1:**
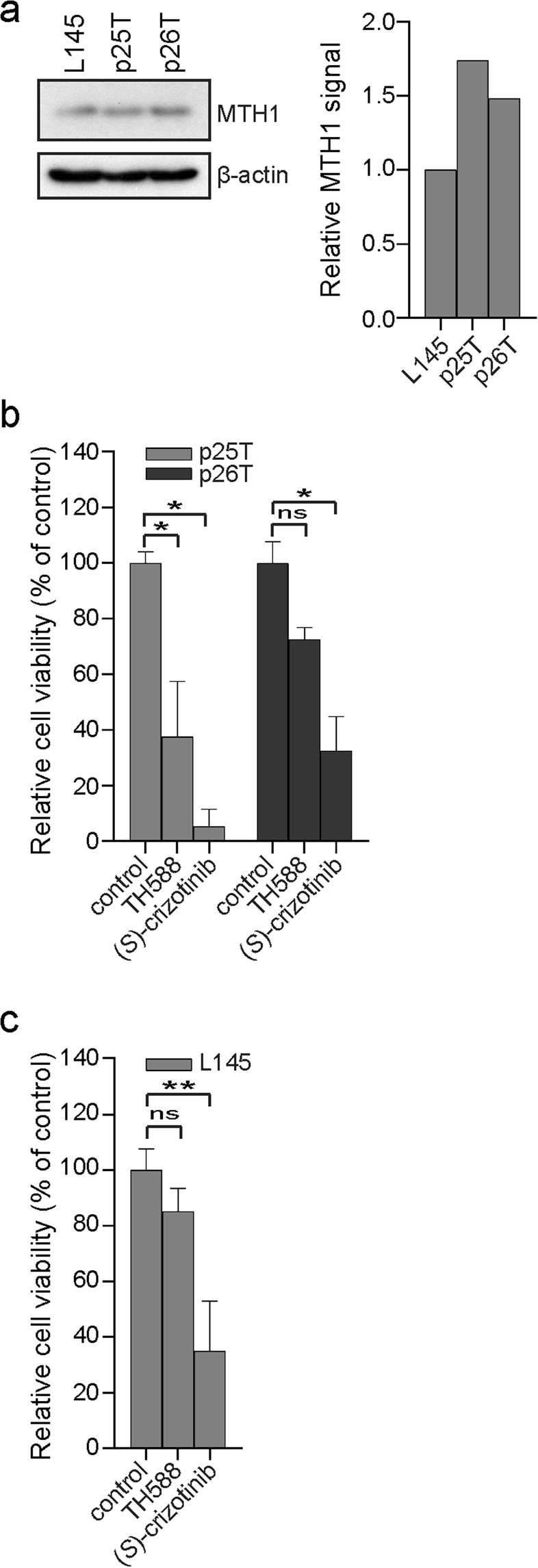
TH588 and (*S*)-crizotinib reduce cell viability of patient-derived colorectal cancer 3D cultures. (**a**) Western blot showing protein levels of MTH1 in L145, p25T and p26T three-dimensional colorectal cancer cultures (left) and the quantification (right). (**b**) Analysis of cell viability of p25T and p26T patient-derived organoid lines after a 3 d treatment with 5 μM TH588 or 5 μM (*S*)-crizotinib. Graph shows mean + s.d. and represents data from two independent experiments. (**c**) Analysis of cell viability of L145 CRC spheroids after a 3 d treatment with 10 μM of TH588 or (*S*)-crizotinib. Graph shows mean + s.d. and represents data from three independent experiments. ns, p > 0.05; *p ≤ 0.05; **p ≤ 0.01 (One-way ANOVA Bonferroni adjusted p-value).

As TH588^8^ and (*S*)-crizotinib^9^ may reduce cell viability by targeting MTH1, we first assured that the MTH1 protein is expressed in the three 3D CRC cultures that we selected. The p25T and p26T organoids were chosen because they are generally extremely sensitive, p25T, and very insensitive, p26T, to various forms of chemotherapy. Western blotting showed that MTH1 is expressed in all three CRC cultures (Fig. 1a). Of interest, gene expression data from the colorectal adenocarcinoma (COADREAD) cohort^25^ shows that MTH1 gene expression is about 2-fold higher in CRC tumour tissue compared to normal colorectal tissue (data available from: http://firebrowse.org/viewGene.html?gene=nudt1).

We next assessed cell viability in response to different concentrations of TH588 or (*S*)-crizotinib in p25T and p26T CRC organoids grown in Matrigel (Supplementary Fig. S1) and in cancer spheroids grown in suspension (Supplementary Fig. S1). Sensitivity to both compounds varied largely between the two organoid lines (Supplementary Fig. S1). Exposure to 5 μM TH588 for 3 days decreased cell viability with 77% in p25T organoids and with 20% in p26T CRC organoids (Supplementary Fig. S1). When treated with 5 μM (*S*)-crizotinib, cell viability was decreased with 90% in p25T and with 39% in p26 (Supplementary Fig. S1). Treating CRC spheroids with 20 μM of TH588 could only reduce cell viability about 20% (Supplementary Fig. S1). In contrast, 20 μM of (*S*)-crizotinib induced massive cell death (Supplementary Fig. S1). Given that 20 μM of (*S*)-crizotinib induced massive cell death of CRC spheroids, we concluded that we should use a lower drug concentration during further experiments. Treating CRC spheroids with 10 μM of TH588 reduced cell viability with 15%, whereas 10 μM of (*S*)-crizotinib reduced cell viability with 65% (Supplementary Fig. S1). To compare efficacy of both compounds we used a similar concentration of TH588 and (*S*)-crizotinib during further experiments. Based on these data and the TH588 and (*S*)-crizotinib concentrations used by the original studies^8,9^, we decided to continue our experiments using 5 μM TH588 or (*S*)-crizotinib when performing experiments with CRC organoids and 10 μM of TH588 or (*S*)-crizotinib when performing experiments using CRC spheroids. These concentrations were used unless stated otherwise.

We next exposed p25T and p26T CRC organoid lines and the CRC cancer spheroid line to TH588 or (*S*)-crizotinib for 3 days and subsequently measured cell viability. (*S*)-crizotinib showed a higher capacity in reducing cell viability compared to TH588 in CRC organoids grown in Matrigel (Fig. 1b) and CRC spheroids that are grown in suspension (Fig. 1c).

### Hypoxia and reoxygenation increase sensitivity to (*S*)-crizotinib but not to TH588

As tumour hypoxia and/or reoxygenation lead to increased production of ROS^3^, we reasoned that these conditions could sensitize tumour cells to the MTH1 inhibitors TH588 and (*S*)-crizotinib. We first performed an Amplex Red Hydrogen Peroxide Assay to detect extracellular hydrogen peroxide (H_2_O_2_) as read-out of ROS levels. We showed that exposure of CRC spheroids for 24 h to hypoxia (0.1% oxygen) and pre-culturing under 0.1% of oxygen followed by reoxygenation for 4 h, increases extracellular levels of H_2_O_2_ (Supplementary Fig. S2). In addition, we have recently shown that exposure of L145 CRC spheroids to 0.1% of oxygen for 24 h also induces ROS-dependent DNA damage^26^.

**Figure 2:**
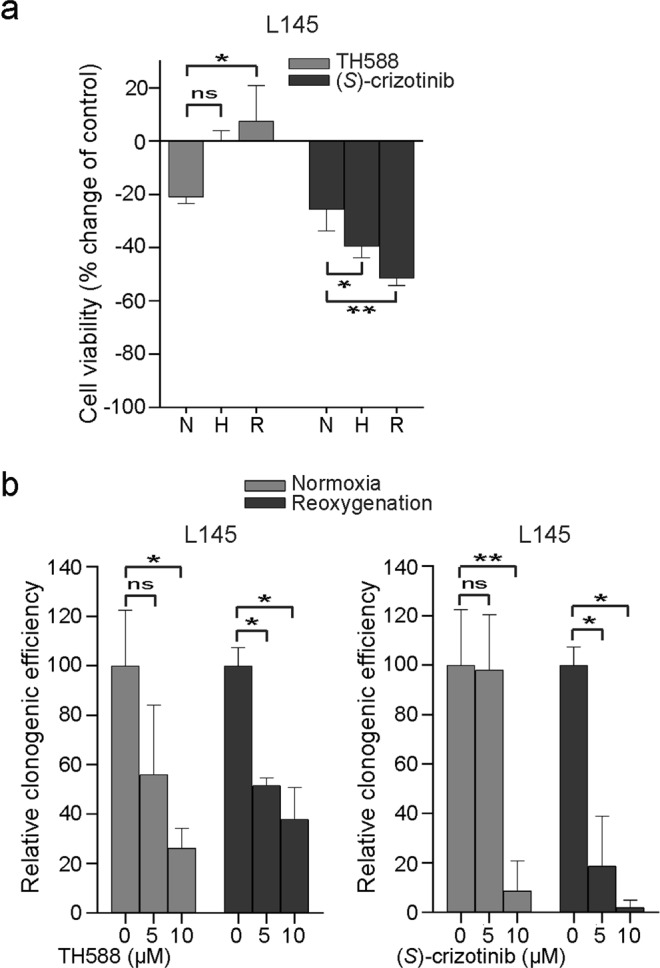
(*S*)-crizotinib sensitivity is enhanced during hypoxia and after reoxygenation whereas colorectal cancer spheroids are not sensitized to TH588 under these conditions. (**a**) Graph showing percentage change in cell viability after a 3 d treatment with 10 μM of TH588 or (*S*)-crizotinib compared to DMSO-treated controls. Human L145 CRC spheroids were cultured under normoxia (21% O_2_) or transferred to a hypoxia chamber (0.1% O_2_). After 72 h, CRC spheroids were either maintained under hypoxia (hypoxia) or returned to normoxia (reoxygenation). Graph shows mean + s.d. and represent data from three independent experiments. ns, p > 0.05; *p ≤ 0.05; **p ≤ 0.01 (One-way ANOVA Bonferroni adjusted p-value). (**b**) Clonogenic capacity after treatment with TH588 or (*S*)-crizotinib (5 or 10 μM). L145 CRC spheroids were cultured as in (**a**). Graphs show mean + s.d. (n = 3 normoxia/n = 2 reoxygenation). ns, p > 0.05; *p ≤ 0.05; **p ≤ 0.01 (ANOVA Bonferroni adjusted p-value). H, hypoxia; N, normoxia; R, reoxygenation.

We next exposed CRC spheroids to hypoxia and reoxygenation in the presence or absence of TH588 or (*S*)-crizotinib. Surprisingly, exposure to 0.1% oxygen reduced (rather than increased) sensitivity to TH588 (Fig. 2a). In addition, sensitivity to TH588 was significantly lower (p = 0.013) when spheroids were reoxygenated just prior to drug exposure (Fig. 2a). In contrast, hypoxia and reoxygenation rendered CRC spheroids significantly (H: p = 0.013; R: p = 0.003) more sensitive to (*S*)-crizotinib (Fig. 2a). It is important to note that late passages of the CRC spheroids were more sensitive to (*S*)-crizotinib, explaining the inconsistencies in viability reduction when comparing the results of Figs 1c and 2a.

In addition, we also performed a cell viability assay under 5% of oxygen. Of interest, the results for treatment with TH588 were in line with the data generated under 0.1% of oxygen (data not shown). However, in contrast to 0.1% oxygen, 5% oxygen did not increase sensitivity to (*S*)-crizotinib whereas reoxygenation did increase sensitivity compared to normoxic culture conditions (data not shown). These results suggest that only reoxygenation after culturing under 5% oxygen increases ROS signalling and therefore might explain the differences in sensitivity to (*S*)-crizotinib under the 5% versus 0.1% of oxygen culture conditions.

We next assessed how TH588 and (*S*)-crizotinib influence the regenerative capacity of human CRC cells. Treatment with 10 μM of TH588 or (*S*)-crizotinib for 3 days significantly reduced clonogenic capacity (TH588: p = 0.016; (*S*)-crizotinib: p = 0.004), whereas 5 μM of either drug had no significant effects (Fig. 2b). To further increase ROS levels, cells were exposed to hypoxia and reoxygenated prior to the addition of the drugs. Reoxygenation enhanced the effect of (*S*)-crizotinib on clonogenic efficiency (Fig. 2b). In contrast, clonogenic efficiency was not further reduced when spheroids were reoxygenated prior to treatment with TH588 (Fig. 2b). These results suggest that TH588 and (*S*)-crizotinib act by targeting different signalling pathways.

### TH588 induces a mitotic arrest in human CRC spheroids

**Figure 3:**
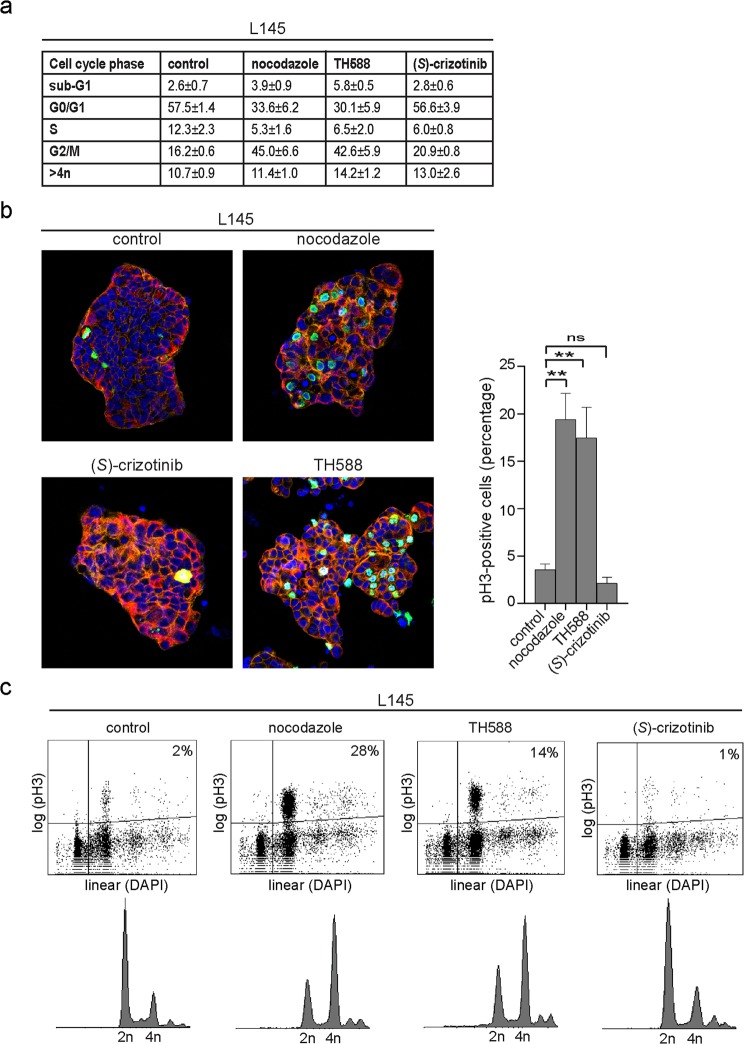
TH588 induces a mitotic arrest in human CRC spheroids. (**a**) Table showing the distribution of cells at the various phases of the cell cycle as determined by flow cytometry using DAPI staining. The values represent the mean percentage + s.d. and represent data from three independent experiments. (**b**) Immunofluorescent staining of the mitotic marker phospho-histone H3 (green), tubulin (red), F-actin (orange) and DAPI (blue) (left) and quantification of phospho-histone H3-positive cells (right). One representative Z-stack per condition is shown. A minimal of 1300 nuclei per condition were analysed. Graph shows mean + s.e.m. ns, p > 0.05; **p ≤ 0.01 (One-way ANOVA Bonferroni adjusted p-value) (**c**) Flow cytometry analysis of DNA content (DAPI staining) and mitotic cell population (phospho-histone H3) of CRC spheroids after exposure to the indicated drugs or DMSO (control) for 1 d. Representative flow cytometry plots are shown (logarithmic scale). Percentages per cell cycle phase are presented in (**a**). (**a–c**) All experiments were performed using L145 CRC spheroids. The following drug concentrations were used: 0.83 μM nocodazole, 10 μM TH588, 10 μM (*S*)-crizotinib. pH3, phospho-histone H3.

We noted that TH588-treated CRC cells adopted a rounded morphology, which could reflect a mitotic arrest^16^. Indeed, analysis of cell cycle distribution by flow cytometry showed that exposure to TH588 for 1 day induced a pronounced G2/M arrest similar to the microtubule-destabilizing agent nocodazole (Fig. 3a and Supplementary Fig. S3). Exposure to (*S*)-crizotinib for 1 day did not increase the population of cells in the G2/M phase (Fig. 3a). During mitosis histone H3 is phosphorylated at serine 10 whereas upon exit of mitosis dephosphorylation of histone H3 takes place^27^. Phospho-Histone H3 (pH3) is therefore a selective marker for mitotic cells^28^. Immunofluorescence analysis of pH3 showed a 5-fold significant increase of pH3 positive cells after treatment with TH588 (p = 0.001) or nocodazole (p = 0.001) (Fig. 3b). By contrast, (*S*)-crizotinib reduced the number of pH3 positive cells (Fig. 3b). In addition, flow cytometry showed that TH588- and nocodazole-treated cancer cell populations had increased percentages of pH3-positive cells among the 4n cell population (Fig. 3c). Finally, TH588 and nocodazole also caused an accumulation of pH3 signal in the p25T and p26T CRC organoid cultures (Supplementary Fig. S4).

### (*S*)-crizotinib inhibits c-MET and ErbB3 in human CRC spheroids

**Figure 4:**
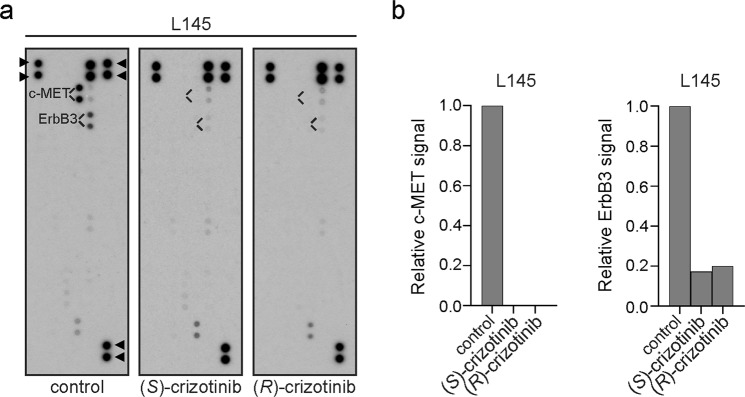
c-MET and ErbB3 phosphorylation is inhibited by (*S*)-crizotinib. (**a**) Detection of phospho-receptor tyrosine kinases in L145 CRC spheroids DMSO-treated (control) or exposed to 10 μM of (*S*)-crizotinib or 10 μM of (*R*)-crizotinib overnight as positive control. 300 μg of lysate was run on each array. Data shown are from a 10 min exposure to film. In the array, each RTK is spotted in duplicate. ► control and reference spots (**b**) Quantification of (**a**).

The (*R*)-enantiomer of (*S*)-crizotinib is clinically used as a dual c-MET/anaplastic lymphoma kinase (ALK) inhibitor^20^. (*S*)-crizotinib also inhibits c-MET, although less potently^20^. By applying a human phospho-receptor tyrosine kinase (RTK) array we compared the effects of (*S*)- and (*R*)-crizotinib on tyrosine kinase receptor phosphorylation in CRC spheroids. We found that overnight incubation with (*S*)-crizotinib or (*R*)-crizotinib reduced phosphorylation of the two most prominently phosphorylated RTKs in CRC spheroids, c-MET and ErbB3, to nearly-undetectable levels (Fig. 4).

### Modulation of ROS levels does not alter the effects of TH588 and (*S*)-crizotinib in CRC cultures

The above findings suggest that TH588 may primarily act as a mitotic spindle poison, while (*S*)-crizotinib is an efficient inhibitor of RTK signalling. To further study the ROS dependency of these distinct treatment effects we made use of the ROS-scavenger N-acetyl-L-cysteine (NAC) and the ROS-inducing compounds menadione or L-buthionine-S,R-sulfoximine (BSO) in combination with auranofin (AUR), an inhibitor of thioredoxin reductase.

We first showed that menadione and BSO/AUR increase over time the extracellular levels of H_2_O_2_ in our 3D model systems (Supplementary Fig. S2). However, we were not able to show that a sublethal dose of BSO/AUR increases extracellular H_2_O_2_ levels in CRC spheroids. Apparently, these concentrations are insufficient to lead to measurable levels of extracellular ROS. The concentration of NAC used during experiments is based on its ability to increase clone-forming efficiency of our CRC 3D lines after seeding single cells in Matrigel, a procedure that will probably induce oxidative signalling.

**Figure 5:**
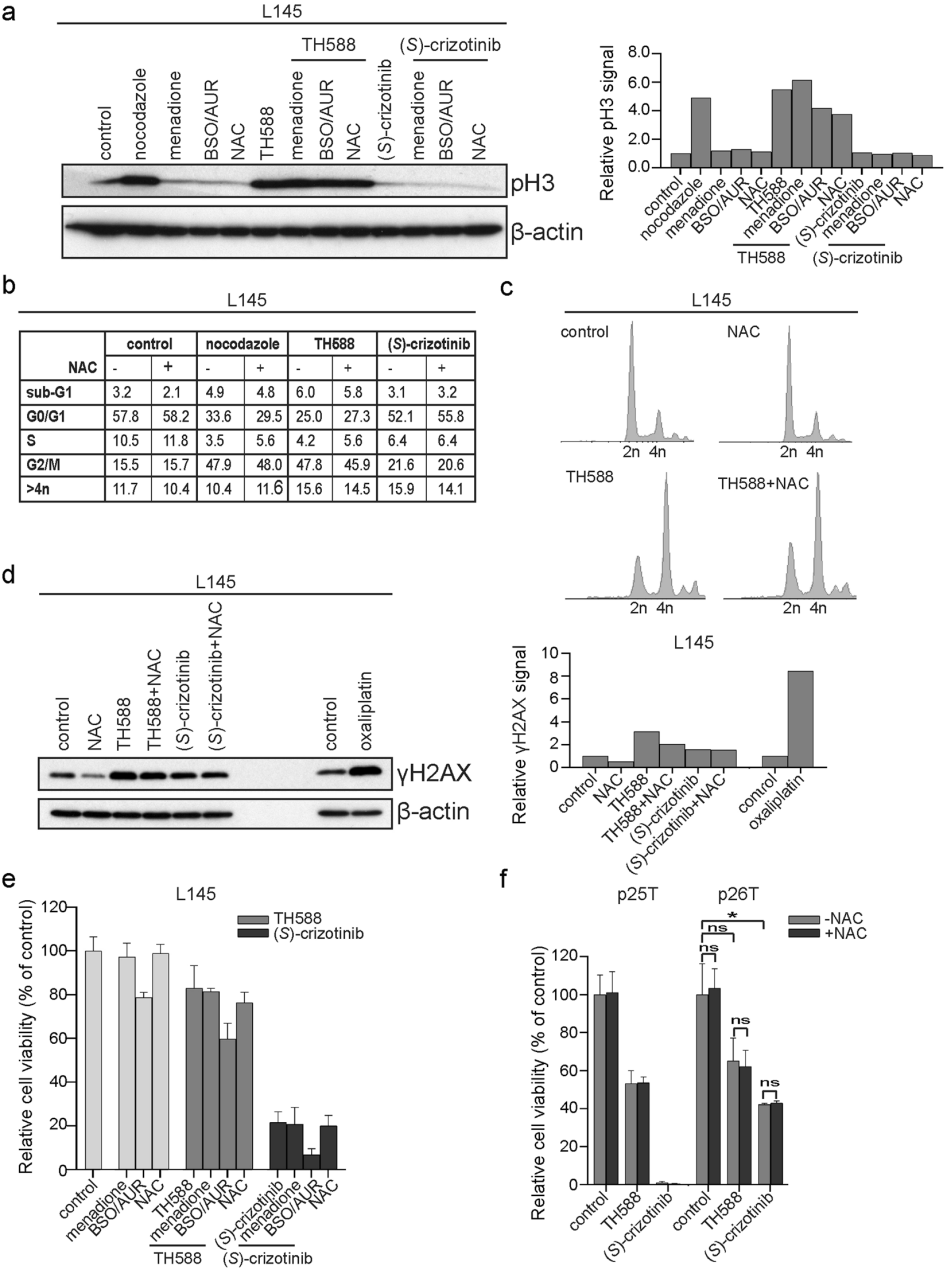
ROS-modulating compounds do not alter sensitivity to TH588 and (*S*)-crizotinib. (**a**) Western blot analysis of phospho-histone H3 protein levels in L145 CRC spheroids following 1d treatment with TH588 or (*S*)-crizotinib combined with the ROS-scavenging compound NAC or ROS-inducing compounds BSO/AUR or menadione, added 2 h prior, 5 h prior, or added during the last 5 h of incubation with TH588 or (*S*)-crizotinib, respectively. Uncropped images are provided in the Supplementary Data. Graph shows the quantification of the Western blot data. (**b**) Table showing the distribution of L145 cells at the various phases of the cell cycle after 1 d treatment as determined by flow cytometry using DAPI staining (n = 1). Some data (conditions without NAC) of Fig. 3a was reused in this table. (**c**) Flow cytometry plots of DAPI staining of L145 spheroids DMSO-treated or exposed to TH588 in the presence or absence of 0.5 mM NAC (logarithmic scale). (**d**) Western blot analysis of γH2AX levels following treatment with TH588 or (*S*)-crizotinib (1 d) in the presence or absence of NAC. L145 CRC spheroids exposed to two cycles of oxaliplatin were used as positive control (n = 1). Graph shows the quantification of the western blot data. (**e**) Analysis of L145 CRC spheroid viability following treatment with TH588 or (*S*)-crizotinib in the ± ROS-modulators. Graph shows mean + s.d. of three replicates (n = 1). (**a–e**) The following drug concentrations were used: 0.83 μM nocodazole, 25 μM menadione, 100 μM BSO, 1 μM AUR, 0.5 mM NAC, 10 μM TH588, 10 μM (*S*)-crizotinib, 2 cycles of 2.5 μM oxaliplatin. (**f**) Analysis of cell viability of organoid cultures after a 3 d treatment with 5 μM of TH588 or (*S*)-crizotinib in the presence or absence of 0.5 mM NAC. Graph shows mean + s.d. of three replicates (p25T) or mean + s.d. from two independent experiments (p26T). Cell viability in the presence of NAC was compared to without NAC for control, TH588- and (*S*)-crizotinib-treated p26T organoids and treatment was compared to DMSO-treated controls using a one-way ANOVA followed by Bonferroni’s post-hoc comparisons test. AUR, auranofin; BSO, L-buthionine-S,R-sulfoximine; NAC, N-acetyl-L-cysteine; pH3, phospho-histone H3; γH2AX, phospho-histone H2AX.

We next exposed CRC spheroids to NAC 2 h prior to treatment with TH588 and analysed pH3 levels as an indicator of mitotic arrest. We found that NAC co-treatment had only a modest effect on TH588-induced pH3 levels (Fig. 5a). Vice versa, co-treatment of TH588 with BSO/AUR (added 5 h prior to treatment) did not result in an increased accumulation of pH3 (Fig. 5a). Combining TH588 with menadione (5 h) only slightly increased pH3 levels (Fig. 5a).

Next, we performed flow cytometry analysis of DAPI and pH3 in CRC spheroids after treatment with TH588 in the absence or presence of NAC. NAC treatment alone did not affect cell cycle distribution (Fig. 5b,c), nor did it affect the G2/M arrest induced by TH588 (Fig. 5b,c).

The generic DNA damage marker phospho-histone H2AX^29^ (γH2AX), indicative of DNA fragmentation during apoptosis, was increased 1 day after incubation with TH588 or (*S*)-crizotinib (Fig. 5d), but could not be rescued by the addition of NAC (Fig. 5d). NAC co-treatment only modestly rescued the γH2AX signal (Fig. 5d). CRC spheroids exposed to 2 cycles of oxaliplatin were added as positive control (Fig. 5d).

The reduction in cell viability caused by TH588 and/or (*S*)-crizotinib could not be rescued by adding the antioxidant EUK-134 (data not shown). In addition, co-treatment with the ROS-modulating compounds menadione, BSO/AUR and NAC did not affect the reduction in viability of CRC spheroids and CRC organoids in response to TH588 or (*S*)-crizotinib (Fig. 5e and Supplementary Fig. S5). The combination of BSO and AUR reduced the cell viability about 20%. This 20% in reduction in cell viability caused by BSO/AUR was still observed when combined with TH588 or (*S*)-crizotinib. This points to an additive effect (and thus different mechanisms) of BSO/AUR and the MTH1 inhibitors in reducing cell viability. Moreover, pre-treatment of CRC organoid cultures with the antioxidant NAC did not rescue the reduction in cell viability induced by either TH588 or (*S*)-crizotinib (Fig. 5f).

## Discussion

In the present manuscript we assessed the potential value of the MTH1 inhibitors TH588 and (*S*)-crizotinib in the treatment of patient-derived 3D CRC cultures by head-to-head comparison under normoxia and hypoxia. Hypoxia is common in solid tumours and may alter the response of tumour cells to MTH1 inhibitors, given its impact on redox signalling. We show that both TH588 and (*S*)-crizotinib have clear but distinct anti-cancer effects in these cultures. TH588 induced a ROS-independent mitotic arrest, which is most likely due to its capacity to inhibit tubulin polymerization and thereby interferes with mitotic spindle assembly^16^. By contrast, (*S*)-crizotinib potently inhibited c-MET and ErbB3 phosphorylation, similar to its (*R*)-enantiomer. Thus, while the MTH1-binding capacity of both drugs is not in question^8,9,15,16,19,30^, the effects on CRC cell survival do not seem to be due to inhibition of MTH1, as they are unaffected by experimental manipulation of ROS levels. Our results obtained in 3D model systems are in line with previous work in other model systems and other cancer types showing MTH1-independent anti-tumour effects of TH588 and (*S*)-crizotinib^15–19^.

However, TH588-induced anti-tumour effects may also be MTH1-dependent^8^, creating an apparent paradox in the literature on the role of MTH1 as valid target for therapy, and the use of its inhibitors in cancer treatment^15–19^ as reviewed elsewhere^14^. Possibly, differences in the extent of ROS production between cancer models may underlie the different results. Indeed, RAS-transformed breast cancer cells that produce high ROS levels depend on continued MTH1 expression for sustained proliferation^31^, in line with the notion that MTH1 could be a valid target in cancer cells that sustain high levels of oxidative stress. This idea prompted us to study MTH1 inhibition as a strategy to kill cancer cells that were exposed to hypoxia and reoxygenation, conditions that occur in solid tumours (as reviewed by^32–34^). We found that hypoxia and/or reoxygenation did not increase the sensitivity of CRC spheroids to TH588. By contrast, loss of the Von-Hippel Lindau factor (VHL) (which degrades hypoxia-inducible factors, HIF1 and HIF2) and/or chemically induced over-activation of HIF1α signalling sensitized zebrafish embryos to TH588, and this was neutralized by pre-treatment with NAC, suggesting that the aberrant redox environment in hypoxic embryos caused sensitization to MTH1 inhibition^35^. Clearly, these results are not in line with the results we obtained in 3D CRC cultures. This may be due to the completely different model systems used, both with respect to the tissues studied (zebrafish embryos *versus* CRC spheroids) and the method used to induce hypoxia signalling (VHL deletion and chemical HIF activation *versus* real hypoxia-reoxygenation). Rather than promoting oxidative damage we found that TH588 induced a mitotic arrest, which is in line with a recent study in HeLa cells^16^. In addition, we showed that co-treatment with ROS-modulating compounds did not influence the anti-proliferative effects of TH588.

Our data further demonstrate that (*S*)-crizotinib reduced phosphorylation of the receptor tyrosine kinases c-MET and ErbB3. Our finding that (*S*)-crizotinib decreases ErB3 activity is in contrast with the original study^9^. However, c-MET inhibitory activity by (*S*)-crizotinib was previously reported^9,20^. Interestingly, we observed a reduction in the number of pH3-positive cells after treatment with (*S*)-crizotinib. The increased sensitivity of CRC spheroids to (*S*)-crizotinib under hypoxia and after reoxygenation could be due to increased or altered signalling of c-MET in response to ROS. The mechanism underlying hypoxia- and reoxygenation-enhanced c-MET signalling might involve oxidation of the protein tyrosine phosphatase (PTP) DEP1^36^, which is a c-MET phosphatase^37^. The available data suggest a model in which hypoxic/reoxygenated cells become increasingly reliant on c-MET signalling, which is stimulated under these conditions by oxidation-dependent inhibition of PTPs such as DEP1.

Regarding mutational status and differences in sensitivity to TH588 and (*S*)-crizotinib, all four patient-derived 3D cultures carry APC mutations and all four have a mutated RAS/RAF pathway with mutations in KRAS, NRAS or BRAF. Except for p7T all cultures have mutated tumour protein p53 (TP53). p25T in particular appears to be most sensitive to treatment, However, the mutations in main driver pathway genes in p25T (i.e. mutations in APC, TP53 and NRAS) does not provide direct clues as to a potential cause of its marked sensitivity. Indeed, it has been very difficult to couple genotype to observed drug responses in organoids, or in the clinic. There was no clear correlation between microtubule-targeting drugs and a specific genotype^38^. Exceptions are the resistance of organoids with mutations in the RAS/RAF pathway and sensitivity to EGF receptor-targeted therapy, the sensitivity of mutant TP53 organoids to nutlin-3a and the sensitivity of WNT ligand-dependent tumours (wildtype APC and mutant RNF43) to porcupine inhibitors^38^.

In conclusion, our results show that TH588 and (*S*)-crizotinib have anti-CRC effects that seem to be not affected by ROS levels and are most likely due to interfering with mitosis (TH588) and RTK activity (*S*)-crizotinib). Multiple microtubule-interacting drugs are used in the standard treatment of various types of cancer, but not CRC. Whether or not TH588, or compounds based on the TH588 structure, have value in comparison to these approved drugs, in particular with regards to their efficacy and toxicity profile, requires further studies. Interestingly, anti-mitotic compounds are generally less effective in targeting hypoxic cells, presumably due to the lower proliferation rate of such cells^39^. Finally, if (*S*)-crizotinib primarily acts as an RTK inhibitor, the added value of this compound compared to its clinically approved (*R*)-enantiomer would be questionable.

Further research should therefore focus on identification of drugs that can exploit increased ROS levels in hypoxic and reoxygenated CRC cells, for instance by using patient-derived 3D model systems. Ultimately, this approach should lead to the development of effective redox-based anti-cancer treatments that can prevent the outgrowth of residual aggressive tumour cells, thereby improving prognosis.

## Methods

### Patient-derived 3D colorectal cancer cultures

Human spheroid cell line L145 was derived from a liver metastasis. Information regarding the mutational status of this CRC spheroid line is provided in Table S1. L145 CRC spheroids are cultured in advanced DMEM/F12 (Gibco) supplemented with 0.5% glucose (Sigma-Aldrich), 100 μM β-mercaptoethanol (Merck), 2 mM Ultraglutamine I (Lonza), trace element A (Cellgro), trace element B (Cellgro), trace element C (Cellgro), 5 mM HEPES buffer (Lonza), 10 μg/ml apotransferrin (Sigma), 20 ug/ml insulin (Sigma), 56 ng/ml progesterone (Sigma), 46.8 ng/mL sodium selenite (Sigma), 1 μg/ml glutathione (Sigma), 8.64 μg/ml putrescin (Sigma), 0.2% lipid mixture 1 (Sigma), 2% antibiotic-antimycotic (Gibco), and 5 μg/ml gentamicin (Life Technologies). 4 ng/ml of fibroblast growth factor (FGF) (PeproTech) was added to the cell culture medium freshly twice a week. This medium will be referred to as cancer stem cell medium (SCM). During experiments, CRC spheroids were cultured in DMEM/F12 (Gibco) in the presence of all cancer SCM additives except for sodium selenite (depleted SCM). Spheroids were cultured in non-tissue culture treated dishes (ThermoScientific). Human organoid lines p7T, p25T and p26T were derived from primary CRC tumours. Genetic information regarding the organoid lines is provided by the original organoid biobank study^38^. Organoids were seeded in droplets of 8 μl each, consisting of 60% Matrigel (Corning) mixed with organoid medium. The composition of organoid medium is: advanced DMEM/F12 (Gibco) supplemented with 1% penicillin-streptocycin (Gibco), 10 mM HEPES buffer (Lonza), 2 mM GlutaMax (Gibco), 2% B27 supplement (Gibco), 1 mM NAC, 100 ng/ml noggin (produced in house), 0.5 μM A8301 (SignalChem), and 10 μM SB202190 (APExBIO). After allowing the Matrigel to solidify, organoid medium was added. During experiments, organoids were cultured in DMEM/F12 (Gibco) in the presence of all organoid medium additives except for NAC and SB202190 (depleted organoid medium). Organoids were cultured in cell-culture treated multiple well plates (Corning). All cell culture was carried out at 37 °C in a 5% CO_2_ humidified incubator under 21% O_2_ (normoxia), 0.1% O_2_ or 5% O_2_ in a Ruskinn Invivo2 hypoxia workstation (The Baker Company) (hypoxia).

### Generation of single cells

Spheroids were made into single cells 3 d before the start of each experiment as previously described^40^. However, 1 mL trypsin was used instead of Accumax and inactivated using medium containing 11% of fetal calf serum (Bodinco BV). CRC spheroids were cultured in SCM and 10 ng/ml FGF was added. p7T, p25T and p26T organoids were made into single cells 2–4 d before the start of each experiment in a similar manner as described for the spheroids elsewhere^40^. However, TrypLE was used for dissociation of the organoids and organoid medium was used to dilute TrypLE after establishment of a single cell suspension.

### Chemicals

TH588 hydrochloride (Axon Medchem), (*S*)-crizotinib (Sigma-Aldrich), and (*R*)-crizotinib (Selleckchem) were dissolved to 10 mM in DMSO for cell experiments. Nocodazole, menadione, BSO, AUR, and NAC were purchased from Sigma-Aldrich. Oxaliplatin was purchased from Selleckchem.

### Western blotting

CRC spheroids were centrifuged and washed twice using PBS. Organoids were first harvested from the Matrigel by incubation with cell recovery solution (Corning) for 1 h on ice. After the Matrigel was dissolved, samples were centrifuged and washed twice using cold PBS. After cells were lysed in buffer containing 120 mM TRIS pH 6.8, 2.5% SDS, and 20% glycerol, samples were boiled for 10–20 min and loaded on 12% or 15% tris-glycine SDS-polyacrylamide gels. Proteins were separated by electrophoresis, and transferred to PVDF membranes, which were blocked in TBST 5% milk (Merck) or in TBST 5% bovine serum albumin (BSA) (Acros Organics) at room temperature (RT) for 1 h. Membranes were probed overnight at 4 °C with primary antibodies anti-NUDT1 (rabbit, Sigma-Aldrich, HPA012636, 1:100) anti-phospho-Histone H3 (Ser10, mouse, Merck, 05–806, 1:1000), anti-γH2AX (Ser139, mouse, Merck, 05–636, 1:1000) or beta-actin antibody (AC-15, mouse, Novus Biologicals, NB600-501, 1:20.000) washed in TBST, incubated with secondary antibodies goat-anti-rabbit (Dako, P0448, 1:1000) and goat-anti-mouse (Dako, P0447, 1:2000) conjugated to horseradish peroxidase at RT for 1 h, and developed using ECL Western Blotting Reagents (GE Healthcare). During processing, images were converted to grayscale and Photoshop levels adjustment was used to improve tonal quality. Uncropped images of all western blot data are provided as Supplementary Data. For quantification, protein signal was measured in ImageJ using the ROI manager tool. Fixed measure areas between samples were used. Signal was corrected for differences in protein loading using the β-actin signal as loading control.

### Cell viability assays

CRC spheroids were seeded in an ultra-low attachment surface 96-well plate, 80 spheroids per well, and cultured in depleted SCM for 3 d. Organoids were first harvested from the Matrigel by incubation with dispase II (Gibco) for 15 min at RT. When the Matrigel was dissolved samples were washed twice using cold PBS. Subsequently, 30 μl of organoid suspension containing 500 organoids and 2% of Matrigel was added to a 96-well plate coated with a mixture of 60% Matrigel and depleted organoid medium. Subsequently, 70 μl of depleted organoid medium was added and maintained at 37 °C. The following day, TH588 or (*S*)-crizotinib was added using a HP D300 Digital Dispenser. After 72 h incubation a CellTiter-Glo Luminescent Cell Viability assay (Promega) was performed according to the manufacturer’s instructions. Luminescence was measured using a luminometer (MicroLumat Plus LB, Berthold Technologies). Luminescence reading was normalized to and expressed as relative percentage of plate-averaged DMSO-treated control.

### Amplex red hydrogen peroxide assay

One part of the CRC spheroids were seeded in an ultra-low attachment surface 96-well plate (5000 spheroids/well) and cultured for 24 h at 37 °C in a 5% CO_2_ humidified incubator under 21% O_2_ (normoxia) or 0.1% O_2_ in a Ruskinn Invivo2 hypoxia workstation (The Baker Company) (hypoxia/reoxygenation) in depleted SCM. To mimic reoxygenation, hypoxic CRC spheroids were cultured under 21% O_2_ for 4 h prior to starting the assay. To mimic hypoxia, hypoxic cells were left in the hypoxic incubator until start of the assay. To the other part of the spheroid suspension, 2% of Matrigel was added and this mixture was subsequently added to a 96-well plate coated with a mixture of 60% Matrigel and depleted SCM. Subsequently, 70 μl of depleted SCM was added and maintained at 37 °C. Organoids were first harvested from the Matrigel by incubation with dispase II (Gibco) for 15 min at RT. When the Matrigel was dissolved samples were washed twice using cold PBS. Subsequently, 30 μl of organoid suspension containing 5000 organoids and 2% of Matrigel was added to a 96-well plate coated with a mixture of 60% Matrigel and depleted organoid medium. Subsequently, 70 μl of depleted organoid medium was added and maintained at 37 °C. Matrigel-embedded spheroids and the organoids were exposed to ROS-modulating compounds for different durations. BSO/AUR and menadione were added 24 h and 2 h before the start of the assay respectively. Medium of the Matrigel-embedded spheroids and organoids was replaced by 50 μl of fresh depleted medium. The Amplex Red Hydrogen Peroxide Assay (Invitrogen, A22188) was performed according to the manufacturer’s instructions. 50 μl of Amplex Red reagent/HRP working solution was added to each well and fluorescence was measured over time at 37 °C. During analyses, the increase in extracellular H_2_O_2_ levels over a period from 10 min to 60 min was compared to the controls.

### Clone forming assay

CRC spheroids were treated for 72 h with TH588 or (*S*)-crizotinib in depleted SCM. After drug incubation spheroids were harvested and made single cells via trypsinisation as described in the section Generation of single cells. Subsequently, 100 viable cells were seeded in droplets of SCM containing 50% Matrigel. Each condition was seeded in triplicate per assay. After polymerization, SCM medium was added supplemented with 4 ng/ml FGF and 0.2 mM NAC. Medium was refreshed twice a week (without the addition of NAC). Two weeks after plating clones were counted.

### Flow cytometry assays

The expression of pH3 was analysed using the BD FACSCelesta (BD Biosciences). CRC spheroids were made into single cells via trypsinisation as described in the section Generation of single cells and fixed in 70% ethanol. Samples were washed twice using PBS containing 0.1% Tween-20 and incubated with the primary antibody anti-phospho-Histone H3 (Ser10, mouse, Merck, 05–806, 1:500) in PBS containing 0.1% Tween-20 at RT for 2 h. Samples were subsequently washed twice and incubated with the secondary antibody goat anti-mouse IgG-Alexa Fluor 488 (Invitrogen, A11029, 1:600) overnight at 4 °C. Samples were washed twice using PBS containing 0.1% Tween-20 and DNA was stained with DAPI (1:500). Data were acquired using a BD FACSCelesta (BD Biosciences) and analysed with BD FACSDiva software version 8.0. Cell debris and doublets were excluded and 10.000 events were measured per experiment.

### Immunofluorescence

Cells were fixed in 4% formaldehyde for 10 min, permeabilized in PBS containing 1% Triton X-100 for 10 min, blocked at RT for 1 h using PBS-Tween 5% BSA, and incubated overnight with primary antibodies dissolved in PBS-Tween 2% BSA at 4 °C. Primary antibodies were anti-tubulin ([YL1/2, rat, Abcam, ab6160, 1:1000) and anti-phospho-Histone H3 (Ser10, mouse, Merck, 05–806, 1:1000). Cells were washed and incubated with Alexa Fluor 568 phalloidin (Life Technologies, A12380, 1:500), secondary antibodies (Alexa Fluor 488 goat anti-mouse, or Alexa Fluor 647 goat anti-rat; Invitrogen) and DNA was stained with DAPI (1:1000) at RT for 1.5 h, and washed twice in PBS-Tween 2% BSA. The final wash was done in PBS. Images (Z-stacks) were acquired using a Zeiss LSM510 Meta Confocal microscope (x40 objective) and analysed with Imaris version 8.2 software (Bitplane AG). ZEN lite 2011 was used to process the data. Individual colour channels for each condition were adjusted to best fit controls.

### Human phospho-RTK array

Spheroids were harvested in cold PBS containing the following phosphatase inhibitors: 25 mM β-glycerophosphate disodium salt hydrate (Sigma-Aldrich), 0.5 mM sodium orthovanadate (Sigma-Aldrich), 0.83 μM okadaic acid (Calbiochem), 1 mM sodium fluoride (Sigma-Aldrich). The human phospho-RTK array (R&D systems, ARY001B) was performed according to the manufacturer’s instructions. Spheroids were harvested in lysis buffer as provided by the manufacturer to which the above-described phosphatase inhibitors were added. A Bradford protein assay was used to determine total protein concentration and 300 μg per sample was loaded. For quantification, protein signal was measured in ImageJ using the ROI manager tool. Fixed measure areas between samples were used. Signal was corrected for differences in protein loading using the reference spots.

### Ethical issues

The collection of tumour tissue for establishment of spheroid lines was approved by the medical ethical committee UMC Utrecht and for organoid lines as described in the original paper^38^. Tumour tissue was obtained in accordance with relevant guidelines and regulations on human experimentation (protocol #09-145 and original study^38^). Regarding the human-derived CRC spheroid and organoid lines^38^, written informed consent was obtained from all participants and/or their legal guardian(s).

### Statistical analyses

Error bars on all graphs show the standard deviation of multiple independent measurements, unless stated otherwise. Statistical analyses were done using IBM SPSS statistics version 25 (IBM corp., Armonk, NY) or GraphPad Prism version 7.0 for Windows (GraphPad Software, La Jolla, California, USA). Planned analyses were performed using a one-way ANOVA followed by Bonferroni’s post-hoc comparisons test. P values < 0.05 (two-tailed) were considered statistically significant.

## Supplementary information

Supplementary Information

Supplementary Table 1

## Data Availability

The data that support the findings of this study are available from the corresponding author on reasonable request.
