# Supplementary material for: Differential anti-tumour effects of MTH1 inhibitors in patient-derived 3D colorectal cancer cultures
Source: Sci Rep. 2019 Jan 28;9:819. doi: 10.1038/s41598-018-37316-w (PMC6349914; doi:10.1038/s41598-018-37316-w)

Supplementary Information

Differential anti-tumour effects of MTH1 inhibitors in patient-derived 3D colorectal cancer cultures

Lizet M. van der Waals[1], Jamila Laoukili[1], Jennifer M.J. Jongen[1], Danielle A. Raats[1], Inne H.M. Borel Rinkes[1] & Onno Kranenburg[1]*

[1]Laboratory Translational Oncology, UMC Utrecht Cancer Center, Utrecht, The Netherlands.

*Address correspondence to Onno Kranenburg, Ph.D., Laboratory Translational Oncology, UMC Utrecht, PO Box 85500, 3508 GA Utrecht, The Netherlands. E-mail: o.kranenburg@umcutrecht.nl. Phone number: 0031-88-7558632

Supplementary Figure Legends

Supplementary Figure 1. Cell viability after exposure to increasing concentrations of TH588 and (*S*)-crizotinib. Analysis of cell viability of p25T and p26T organoids **(a)** or L145 CRC spheroids **(b)** after a 3 day treatment with increasing concentrations of TH588 or (*S*)-crizotinib. Graphs shows mean + s.d. for three replicates **(a)** or mean + s.d. of two independent experiments **(b).**

Supplementary Figure 2. Hydrogen Peroxide levels. Extracellular H_2O_2 levels were measured over time using an Amplex Red Hydrogen Peroxide Assay in L145 CRC spheroids **(a,b)**, p7T CRC organoids **(c)** or p26T organoids **(d)**. CRC spheroids were cultured under normoxia, hypoxia or reoxygenated **(a)** or cultured in the presence of ROS-modulators **(b)**. **(c,d)** CRC organoids were cultured in the presence of ROS-modulators. **(a-d)** Graphs show the increase in extracellular hydrogen peroxide levels over time. The following drug concentrations were used: 25 µM menadione, 100 µM BSO (or 10 µM), 1 µM AUR (or 0.1 µM).

Supplementary Figure 3. Cell cycle profile of TH588-treated L145 CRC spheroids resembles microtubule-targeting agent nocodazole. Graphic representation of figure 3a. Flow cytometry analysis using DAPI was performed on CRC spheroids left untreated (control), or treated with 10 µM TH588, 10 µM (*S*)-crizotinib, or 0.83 µM nocodazole as positive control. The values represent the mean percentage + s.d. and represent data from three independent experiments.

Supplementary Figure 4. TH588 increases levels of phospho-histone H3 positivity in organoid cultures. Western blot showing levels of phospho-histone H3 in p25T and p26T organoids treated with DMSO (control), or treated with 5 µM TH588, 5 µM (*S*)-crizotinib or 0.83 µM nocodazole as positive control for 1 day. pH3, phospho-histone H3.

Supplementary Figure 5. Analysis of viability of p7T and p26T organoid cultures following treatment with TH588 or (*S*)-crizotinib in the presence or absence of ROS modulators. The following drug concentrations were used: 25 µM menadione, 100 µM BSO, 1 µM AUR, 0.5 mM NAC, 5 µM TH588, 5 µM (*S*)-crizotinib. The values represent the mean percentage + s.d. and represent data from two independent experiments.

Supplementary Figure 1

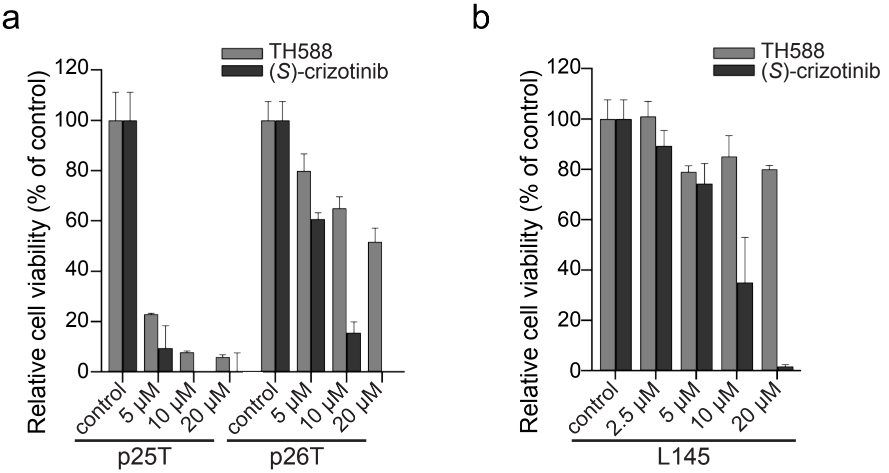

Supplementary Figure 2

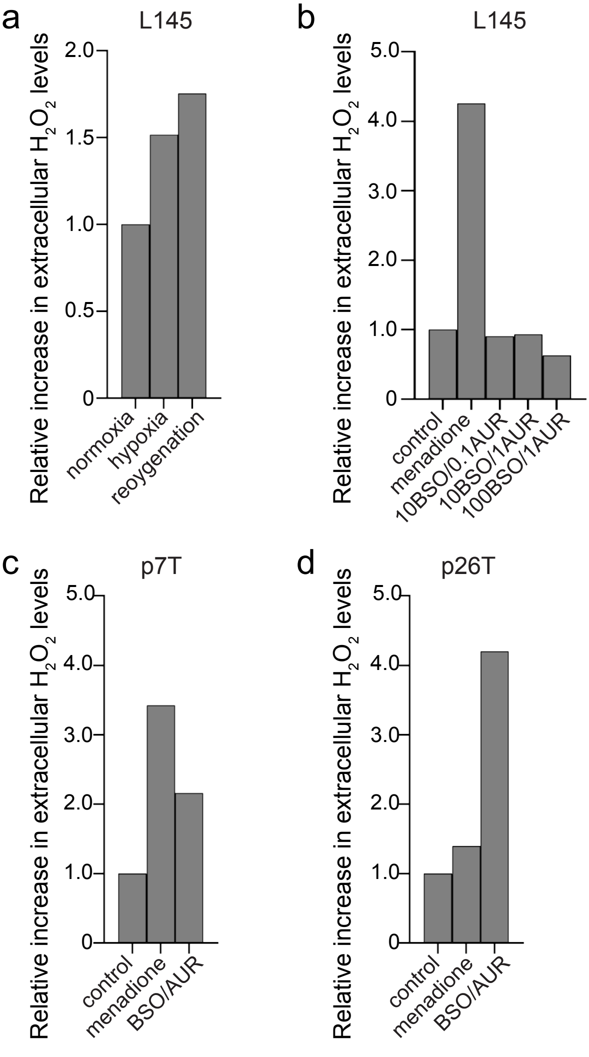

Supplementary Figure 3

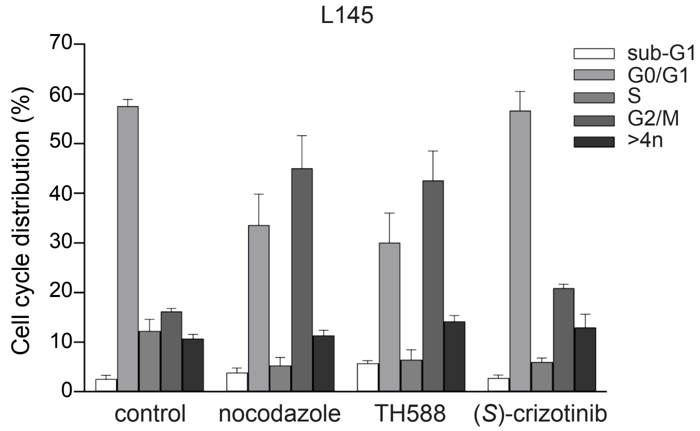

Supplementary Figure 4

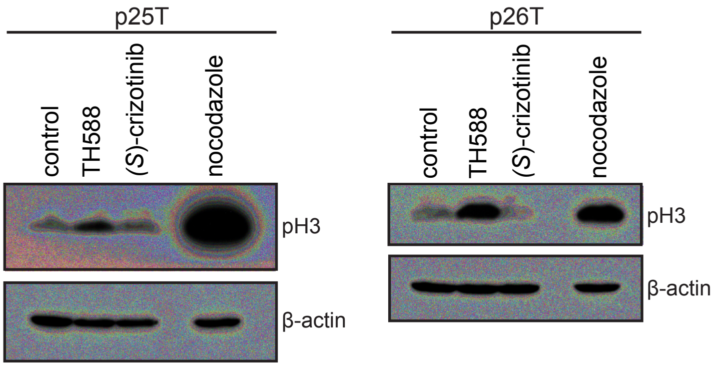

Supplementary Figure 5

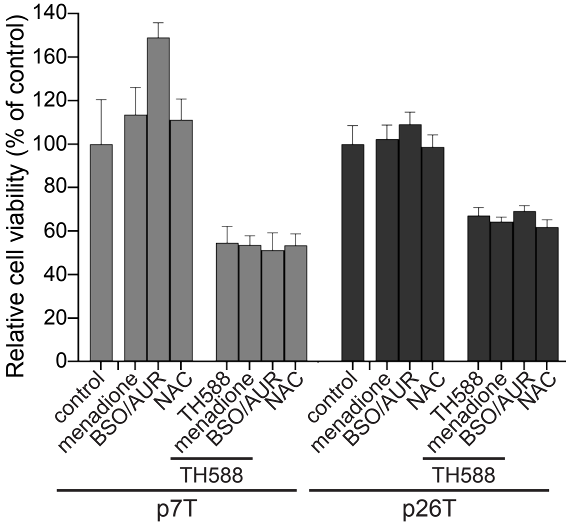

Uncropped images of all the western blot data

Figure 1a

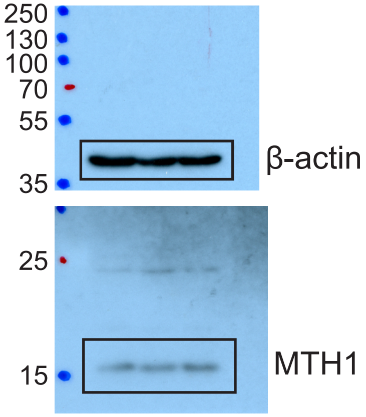

Figure 5a

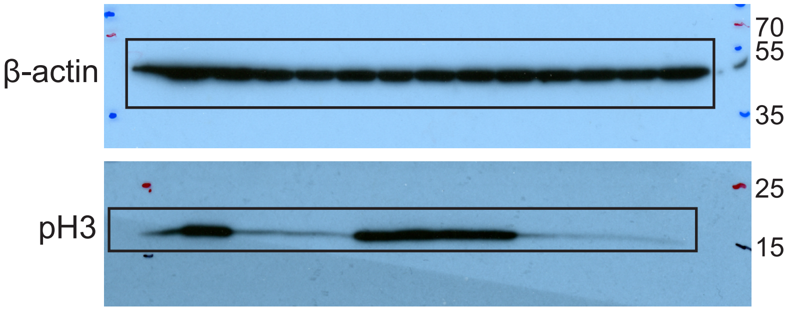

Figure 5d

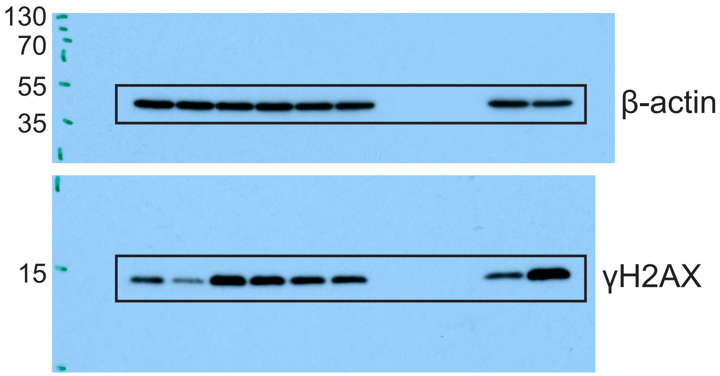

Uncropped images of all the western blot data (continued)

Figure S4

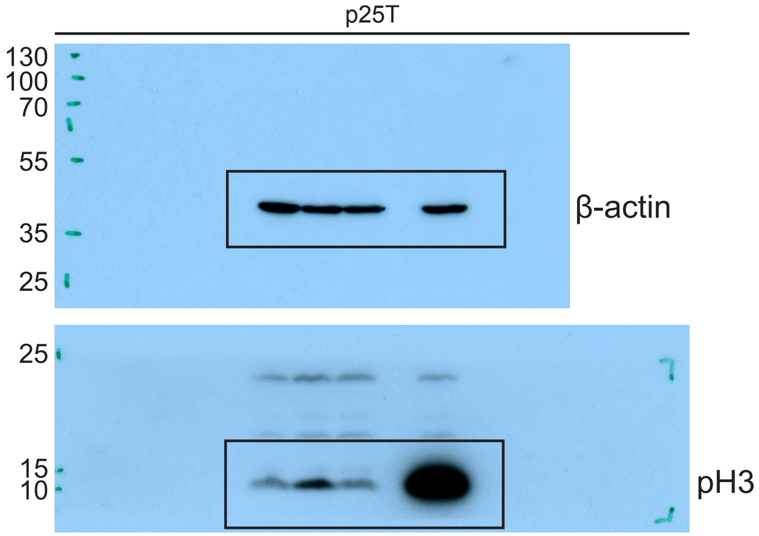

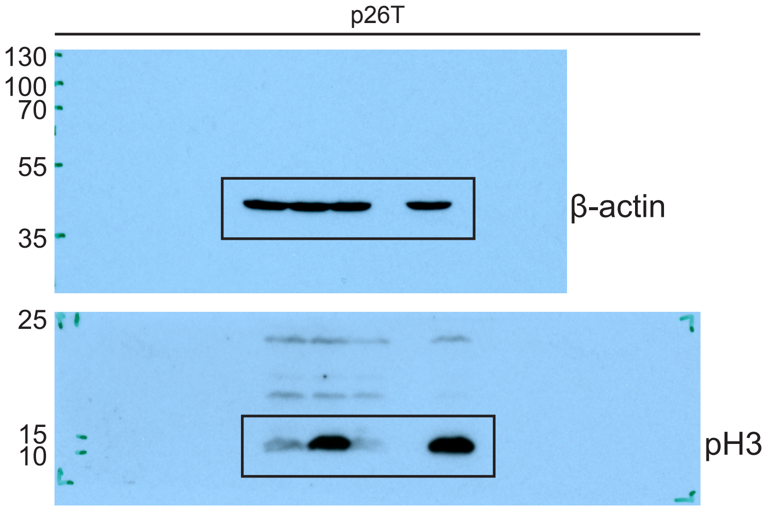

Supplement: Supplementary file 1 — Supplementary Information [file 41598_2018_37316_MOESM1_ESM.pdf]
